# Research on optimal control design of displaced left turn signal at one two way and three one way traffic intersections

**DOI:** 10.1038/s41598-025-90009-z

**Published:** 2025-02-24

**Authors:** Ning Han, Guozhu Cheng, Jiadong Lin, Zhiyun Tang, Fei Xie

**Affiliations:** 1 School of Civil Engineering and Transportation, Northeast Forestry University, Harbin, 150040 China; 2 School of Transportation Science and Engineering, Harbin Institute of Technology, Harbin, 150090 China

**Keywords:** Single-double traffic intersections, One-way traffic, Displaced left-turn, Pre-signals, Engineering, Civil engineering

## Abstract

As the nodes of urban road traffic network, intersections serve as an effective means to improve traffic flow efficiency and alleviate traffic congestion through the optimization of reasonable traffic organization and signal schemes for intersections. In recent years, domestic research on unconventional intersections has attracted increasing attention. This paper first summarized the types of single-double traffic intersections and preliminarily discussed the applicability of displaced left-turn (DLT) signal organization. We then constructed a calculation method for determining the important design parameters in the DLT signal traffic organization, based on the design of the channelization form of the intersection of Haihe Road and Xuanqing Street in Harbin City, China. Based on the average delay of vehicles, we established a signal optimization control model with two components: the main signal delay and the pre-signal delay, aiming to minimize the delay of vehicles. The signal optimization control model was established considering both the main signal delay and the pre-signal delay. Finally, the selected examples were compared and verified using VISSIM simulation. The simulation results show that through the design of one two-way and three one-way traffic intersections with DLT signals and signal optimization control, the average vehicle delay at the intersection is significantly reduced, and traffic efficiency is enhanced.

## Introduction

With the acceleration of urbanization and the growing demand for transportation, traffic congestion has become a major problem for urban development. In the face of increasingly serious traffic problems, the most traditional approach is to build roads to expand the spatial scale of the road network, which can improve the traffic situation to a certain extent, but the available land in the city is limited, and new roads may also induce new traffic demand. How to maximize the use of existing roads, the use of scientific traffic management means to rationally organize traffic flow, to tap the potential capacity, is currently the realization of urban road traffic smooth, safe and more practical methods.

One-way traffic is a type of road traffic that only allows vehicles to travel in a certain direction, and the United States first began to use one-way traffic in 1906 to solve traffic problems in urban areas^1^. Hocherman et al. (1982) have found through the statistical analysis of accident data over several years in Jerusalem that the conversion of two-way traffic to one-way traffic capacity increased by 19%, and vehicle speeds increased by an average of 37%^2^. Persaud et al. (1997) have conducted a study on one-way intersections in Philadelphia to analyze the effects of signal control and stop-and-yield control on intersection crashes, and the results showed that stop-and-yield control resulted in a 24% reduction in crashes compared to signal control^3^. On the basis of the above example analysis and statistics, some scholars have further studied the role and influence mechanism of one-way traffic, and get the relevant theories about signal timing, economy, safety and so on. By analyzing the aggregation effect of turning vehicles on urban traffic networks, Gayah (2012) has explained that the maximum vehicle flow into the roadway network is increased during congestion due to the fact that left-turning vehicles at one-way traffic intersections have fewer conflicts with other streams of traffic, which ultimately leads to more chaotic and inefficient behavior^4^. Guler et al. (2014) have proposed an algorithm based on two one-way streets for networked vehicles at signalized intersections with the goal of minimizing delay, and the results showed that the delay of networked vehicles at intersections can be reduced by up to 60%^5^. In the context of today’s proliferation of vehicles, one-way traffic has been widely used as a traffic management strategy to effectively solve traffic congestion on urban roads.

As an important node of the road network, intersections are crucial for enhancing the traffic efficiency of the whole road network. Urban intersections are often bottleneck sections of urban roads, and in order to alleviate congestion on urban roads, many scholars have studied how to improve the efficiency of intersections from different aspects. In terms of signal control, Huang et al. (2024) have proposed an optimization method for lane assignment and adaptive signal control at isolated intersections under stochastic demand using the concept of level-of-service (LOS) reliability, which can better adapt to the stochastic traffic demand^6^. Sattarzadeh et al. (2024) have integrated latent and implicit Markov models for a network with multiple intersections to develop an adaptive traffic signal control (ATSC) framework, achieving good results in balancing and managing network traffic^7^. With the development of connected vehicles and autonomous driving technologies, some scholars have proposed cooperative control models for traffic signals and CAVs based on CV/CAV trajectory data^8,9^. In traffic management, Sahil et al. (2021) have proposed a real-time traffic control model for intersection traffic flow using IoT technology to improve the safety and efficiency of intersections using IoT technology^10^.

Displaced left-turn (DLT) is an innovative traffic design scheme to eliminate left-turn and straight-traffic conflicts at primary signalized intersections by installing pre-signalized intersections with channelized left-turn lanes shifted in the roadway segments of the intersection inlets^11^. Displaced left-turn intersections were first developed in the United States with Mexico and piloted in some cities^12^. Jagannathan et al. (2004) have conducted a design with guiding values for shifted left-turn lane widths, pre-signalized intersections, and radius of vehicular movement at the main signalized intersections^13^. You et al. (2013) have proposed a method to optimize the phase sequences based on the displaced left-turn Signal intersections, cycle length, and signal interval of a full continuous flow intersection (CFI) model, and the comparison results showed that displaced left-turn Signal intersections have obvious advantages in terms of minimum cycle, capacity, and average vehicle delay^14^. Sun et al. (2015) have proposed a simplified Continuous Flow Intersection (CFI-Lite) design based on the concept of CFI, which does not require a new sub-intersection, but utilizes the upstream intersection to distribute left-turn traffic to the displaced left-turn Lane, which has good results on arterials with close intersection spacing^15^. Zhao et al. (2015) have proposed a lane-based generalized optimization model for DLT intersection types, lane markings, displaced left-turn Signal lengths, and signal cycle integration design, which aims to systematically design DLT intersections and provide traffic professionals with a design basis^16^. Abdelrahman et al. (2020) have conducted a comparative group comparison and cross sectional analysis study on safety and operational efficiency of DLT intersections, DLT intersections increase the probability of collision compared to conventional intersections, but may be more effective for operational performance, and it is recommended that appropriate safety countermeasures be developed and implemented^17^.

Single-Double Traffic Intersections (SDTI) are intersections formed by the intersection of one-way traffic and two-way traffic in areas where one-way traffic management is implemented. By reducing the number of conflict points and intersections, single-double traffic intersections can significantly improve road capacity, organize and manage traffic flow more effectively, and reduce the occurrence of traffic accidents to a certain extent. However, due to the one-way design of the road, the signal control method for single-double traffic intersections may aggravate traffic congestion when there is excessive traffic flow. Similarly, displaced left-turn intersections can significantly improve the capacity of the intersection, but there are some problems, especially the overflow of left-turn queuing vehicles into the intersection, resulting in traffic congestion at the intersection, which can easily paralyze the operation of the intersection.

Therefore, taking into account the advantages and disadvantages of single-double traffic intersections and the problems faced by the displaced left-turn intersections, this paper proposes a one two-way and three one-way traffic intersections displaced left-turn Signal Optimization and Control Design research method. Harbin, the capital of Heilongjiang Province, China, has 180 existing one-way roads, which are mainly concentrated in the central area of the city and have formed block one-way traffic organization. This paper takes Harbin Haihe Road - Xuanqing Street intersection as an example, and proposes a method to set up DLT lanes in two-way roads, and to integrate the design of the pre-signal intersection length, main signal phase sequence, cycle time, and green light time interval between main and pre signals. By displaced left-turn lane organization for this single-double traffic intersections to further reduce the conflict points, it can effectively solve the problems of traffic congestion, excessive queuing, higher traffic delays, etc. Compared with traditional signal design, this method expands the range of intersections. By designing the time and space reasonably within the range of single and two-way intersections, the conflict points within the intersection range are transferred to the road section. Through collaborative control of pre signals, the number of phases is reduced, stopping delays are reduced, and the traffic efficiency of intersections is improved, the effect is significant during peak hours at high-capacity one-way and two-way traffic intersections.

The remaining parts of this paper are arranged as follows: The second part discusses the types of single-double traffic intersections and the applicability of displaced left-turn organization, and proposes a design method for displaced left-turn organization at one two-way and three one-way traffic intersections. The third part models and analyzes design parameters such as the length of the displaced Left-Turn (DLT) lanes, the width of the Pre-signalized intersection, and the control of displaced left-turn signals in the organization of left-turn traffic. The fourth part proposes the displaced left-turn signal optimization control model based on the minimum stopping delay. The fifth part presents a case study of one two-way and three one-way traffic intersection in Harbin, China, and uses VISSM for simulation analysis and evaluation. Finally, the sixth part presents the research conclusions and future research directions.

## Displaced left turn design for single-double traffic intersections

### Analysis of types and applicability of single-double traffic intersections

**Fig. 1:**
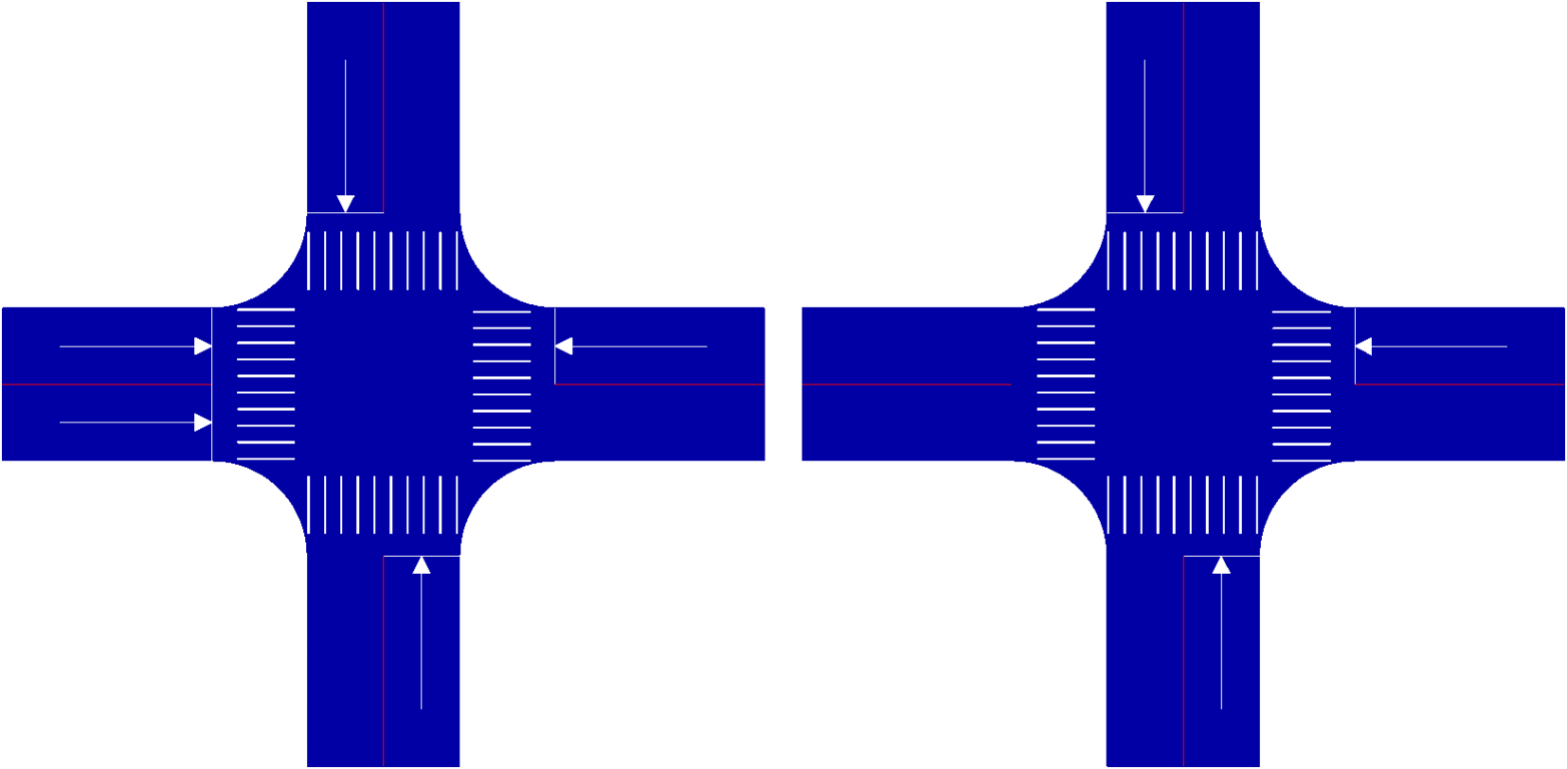
Three Two-way and One One-way Intersections.

Setting up one-way traffic areas will result in single-double traffic intersections, single-double traffic intersections can be divided into three two-way and one one-way, two two-way and two one-way and one two-way and three one-way traffic intersections. Among them, the three two-way and one one-way traffic intersection can take two forms based on its single traffic direction, as shown in Fig. 1.

**Fig. 2:**
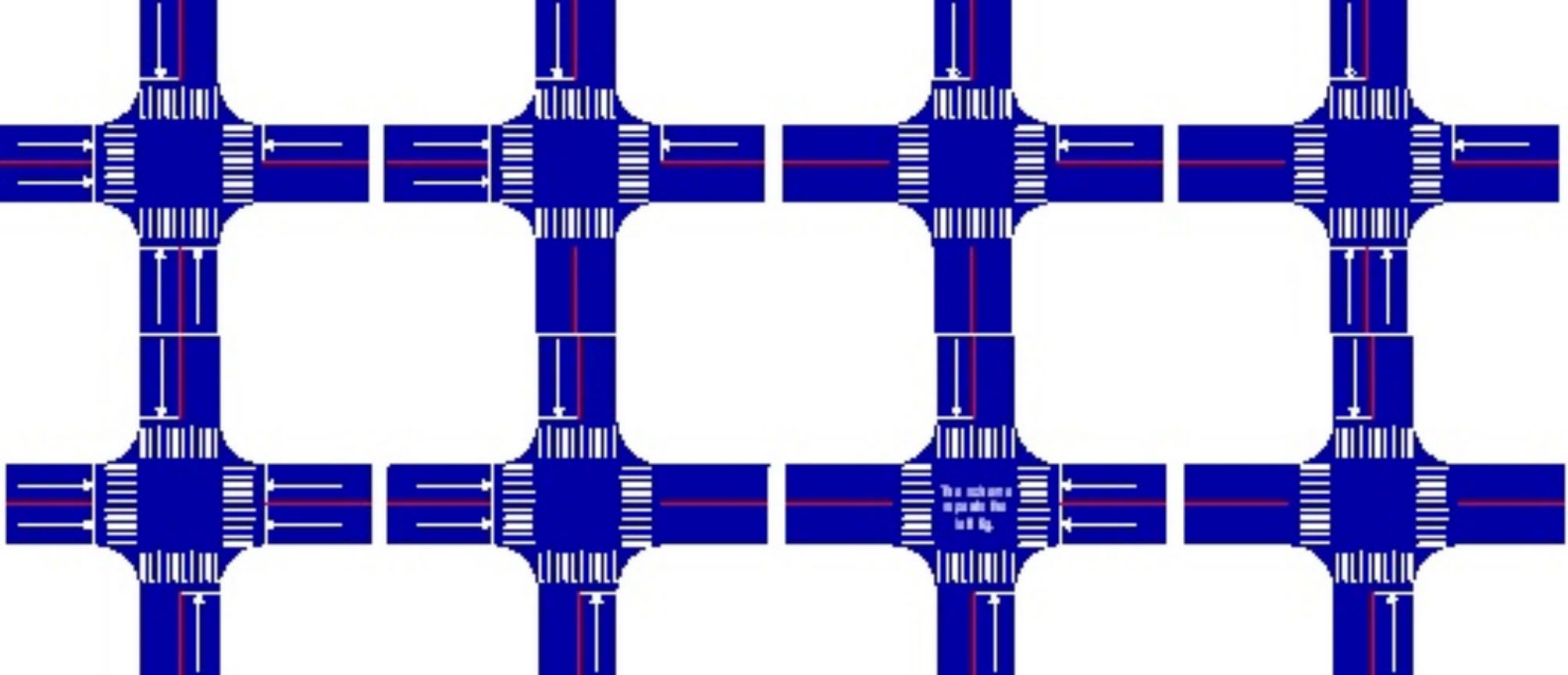
Two Two-way and Two One-way Intersections.

**Fig. 3:**
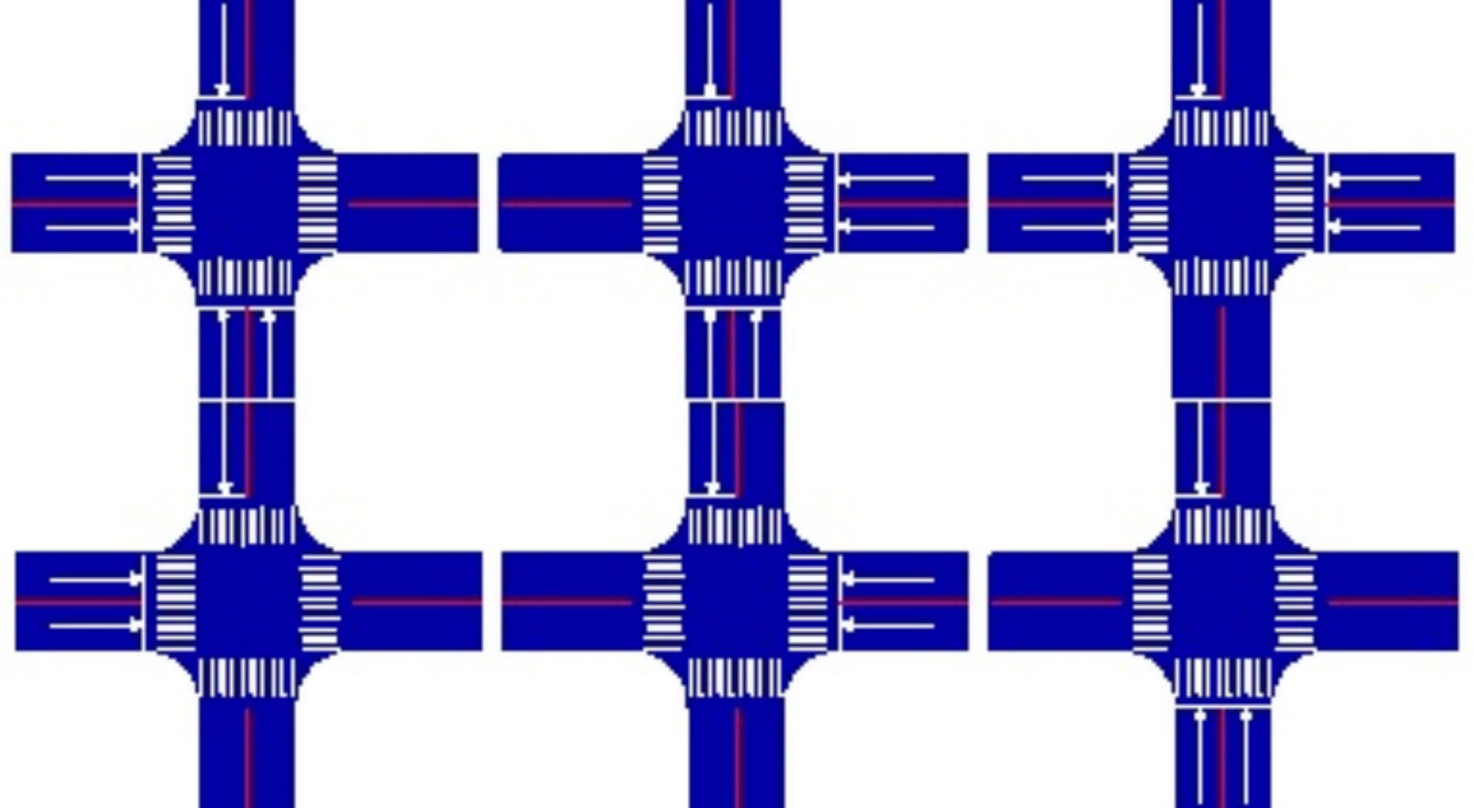
One Two-Way and Three One-Way Intersections.

There are seven forms of two two-way and two one-way traffic intersections based on the relative location of their double traffic and the direction of the single traffic, as shown in Fig. 2. In Fig. 3, there are six forms of one two-way and three one-way traffic intersections based on the relative location and direction of their single traffic.

With the increasing number of directions for one-way traffic organization at single-double traffic intersections, the number of conflict points at intersections gradually decreases. This can significantly improve the traffic efficiency of intersections, but conflict points cannot be completely eliminated. In addition, the vehicle flow and pedestrian flow patterns in different directions at single-double traffic intersections are more complex, often facing problems such as high stopping delays, high collision risks, and long signal cycles.

The single-double traffic intersection adopts a displaced left-turn design, which sets up a pre-signal intersection upstream of the two-way traffic section and sets the left turn lane on the left side of the exit lane, called displaced left turn (DLT). The principle is that left turning vehicles enter the displaced left turn lane at the pre-signal intersection, avoiding conflicts with oncoming vehicles at the main signal intersection, thereby transferring the conflict of the main signal intersection to the pre-signal intersection. Through collaborative control of the pre-signal, stopping delays are reduced and the traffic efficiency of the intersection is improved. Therefore, all single-double traffic intersections with severe left and straight conflicts can be designed for displaced left-turn traffic organization based on their road and traffic conditions.

Three two-way and one one-way traffic intersections implement displaced left-turns in the two-way lanes based on left-turning traffic entering or exiting one-way traffic lanes. This reduces the intermingling of left-turning traffic in their primary direction with other traffic flows.

Two two-way and two one-way traffic intersections require careful consideration of the relative position and direction of the double traffic roads, as well as the traffic flow and direction. The design must be tailored to the specific conditions of the intersection.

One two-way and three one-way traffic intersections implement displaced left-turns for the double traffic road based on the volume of left-turning traffic entering or exiting the two-way traffic section.

One two-way and three one-way traffic intersections have theoretically eliminated 80% of the traffic conflicts and is the simplest form of the single-double traffic intersection. By implementing displaced left-turn traffic organization in the two-way lane section, these conflicts can be further reduced, and the intersection capacity can be increased. Therefore, this paper will systematically study one two-way and three one-way traffic intersections from the following aspects: Displaced left-turn main intersection channelization, Pre-signal intersection width, Displaced left-turn lane length, Main signal and pre-signal phase sequences, Main and pre-signal coordination design. The feasibility of the displaced left-turn organization for one two-way and three one-way traffic intersections will be verified based on actual cases.

### Geometric design of displaced left-turn traffic organization

**Fig. 4:**
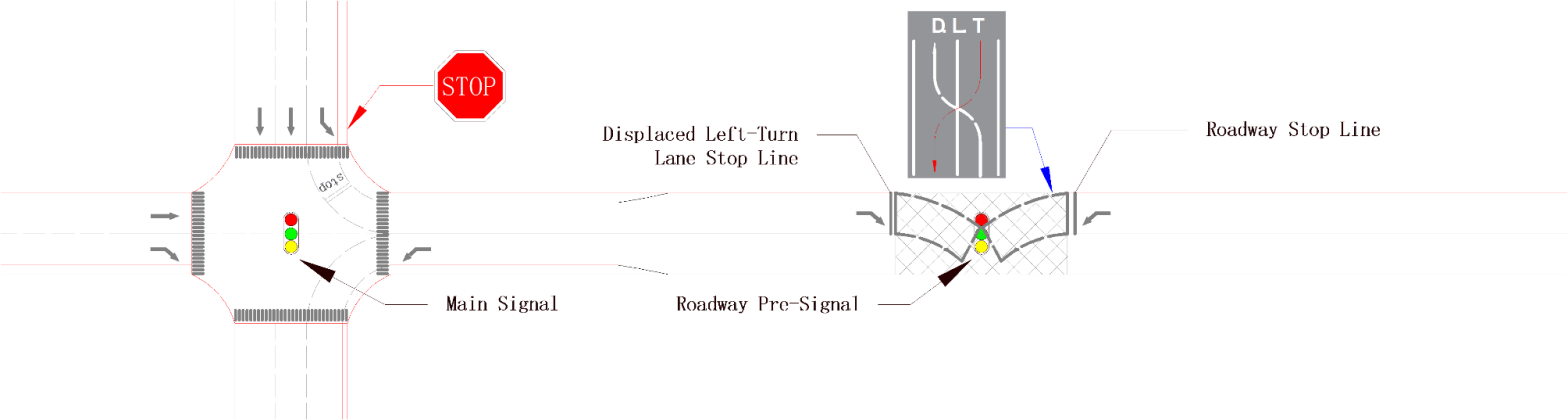
Traffic organization diagram for displaced left-turns.

Take the east inlet for double traffic, with the rest of the direction of the inlet for single traffic in a one two-way and three one-way traffic intersection as an example. The double traffic section of the displaced left-turn design and geometric design are shown in Fig. 4. The specific operation method is as follows: set up a pre-signal intersection in the one two-way and three two-way traffic intersection two-way road that is the upstream of the east inlet road, move the east inlet left-turn lane to the left side of the exit road, set up a stop line and pre-signal at the pre-signal intersection, and set up the displaced left-turn signal guide line. For ease of description, the westbound stop line at the pre-signal intersection is the roadway stop line, and the eastbound stop line is the displaced left-turn lane stop line.

The displaced left-turn signal organization advances the conflict point between the opposing straight and left-turning traffic to be handled at the pre-signalized intersection of the roadway section, reducing the number of conflict points at the main intersection. Through traffic channelization and coordinated control of the main and pre-signals, left-turning traffic and opposing straight traffic are given the right-of-way within the same green time. As a result, the number of phases and the waiting time of the traffic flow are reduced, and the capacity of the intersection is improved.

## Displaced left-turn traffic organization design parameter determination

### Displaced left-turn lane length determination

**Fig. 5:**
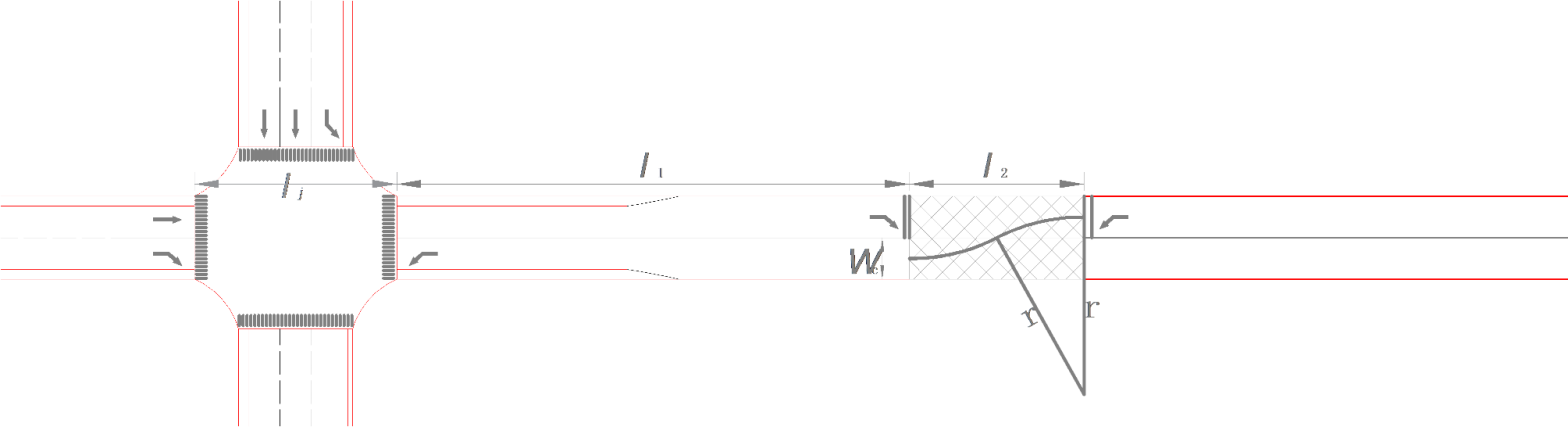
Design parameters for displaced left-turn traffic organization.

Displaced left-turn lanes are installed at the east inlet, and vehicles change lanes through the pre-signal intersection to realize the transition between the displaced left-turn lanes and the lanes in the normal direction of travel. The displaced left-turn lane lengths and pre-signal intersection widths are shown in Fig. 5.

Northbound left-turning vehicles are required to first stop at the displaced left-turn lane stop line and proceed through the pre-signalized intersection under pre-signal control. To avoid the north inlet left-turn vehicles queuing in the displaced left-turn lane from blocking the main intersection, the length of the displaced left-turn lane is related to the maximum queue length of the north inlet left-turn traffic, so the length of the displaced left-turn lane should be satisfied:

1 $${l_1} \geqslant \frac{{{{\bar {q}}_{nl}} \times \vartriangle T \times {h_s}}}{{3600}}$$

Where: *l*_1_ is the length of displaced left-turn lane/m; ${\bar {q}_{nl}}$ is the arrival rate of northbound left-turning vehicles; $\vartriangle T$ is the queuing waiting time of northbound left-turning vehicles in the signal cycle/s; *h*_*s*_ is the headway of north-imported left-turning vehicles in the queue/m.

### Pre-signalized intersection width determination

East inlet displaced left-turn vehicles entering the displaced left-turn lane from the roadway stop line need to transition through the pre-signal intersection. During the lane change transition, the length of the lane change is related to the minimum turning radius of the vehicle. Based on the traffic composition, the minimum turning radius is calculated by selecting the largest vehicle type, i.e., bus (usually taken as 13–13.5 m). Considering the minimum safety distance between front and rear vehicles in the same lane and the minimum safety distance between adjacent lanes, the pre-signalized intersection width should be designed to satisfy the following requirements:

2 $${l_2} \geqslant 2\sqrt {{r^2} - {{(r - \frac{{{w_c}}}{2})}^2}}$$

Where: *l*_2_ is the width of the pre-signal intersection/m; *r* is the minimum turning radius of the vehicle/m; *w*_*c*_ is the width of a lane/m.

### Determination of optimal control method for displaced left-turn signal

#### Phase and phase sequence of main signal and pre-signal

As a result of the displaced left-turn lane, north inlet left-turning vehicles, west inlet right-turning vehicles, and east inlet left-turning vehicles are not controlled by the main intersection signal at the main intersection and may proceed directly through the main intersection. Therefore, the intersection main signal is two-phase, with the north inlet straight-ahead in the first phase and the west inlet straight-ahead in the second phase. The pre-signal at the pre-signal intersection also operates in two phases: the east inlet displaced left-turn as the first phase, the north inlet left-turn and the west inlet straight-ahead as the second phase. The pre-signal is activated to allow east inlet displaced left-turn vehicles to pass, and it is deactivated to allow north inlet left-turn vehicles and west inlet straight-ahead vehicles to pass.

#### Coordination design scheme between main signal and pre signal

**Fig. 6:**
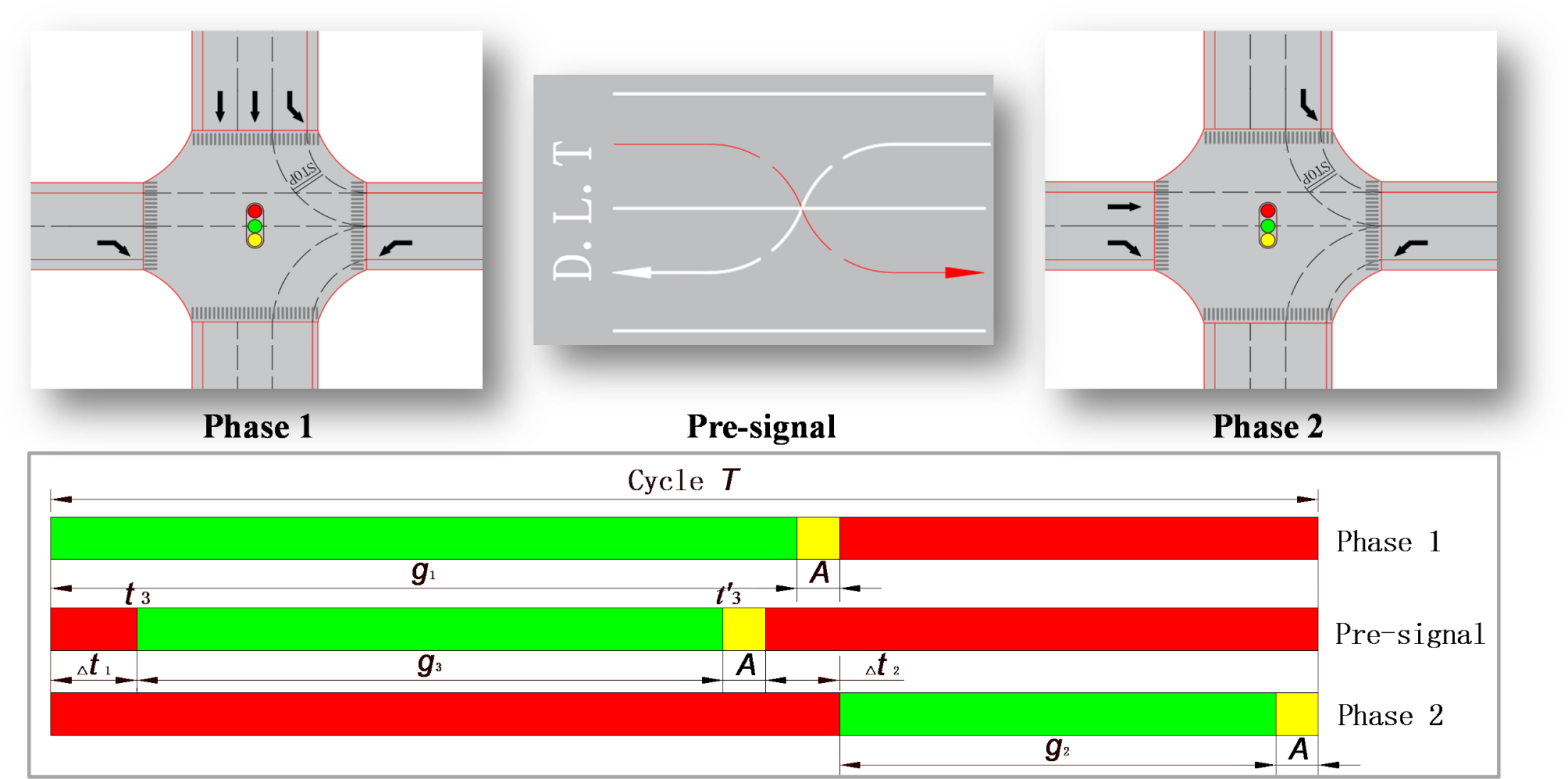
Optimized control design parameters for displaced left-turn signal.

In order to maximize the overall capacity of the intersection, when the roadway pre-signal is on, east inlet displaced left-turn vehicles drive through the roadway stop line and proceed through the intersection continuously. At this time, north inlet arriving left-turn vehicles line up in front of the displaced left-turn lane stop line. When the west inlet straight through traffic passes, the roadway pre-signal is turned off early to allow the north inlet left-turning vehicles to dissipate early so that the west inlet straight through traffic continuously moves out of the pre-signal intersection. In order to improve the intersection operation efficiency and reduce the number of delays and stops of vehicles in all directions, determine the main signal and pre-signal coordination design program, the design parameters are shown in Fig. 6.

I. In order to avoid secondary queuing of north inlet arriving straight through vehicles, all vehicles in the north inlet straight lane should be made to exit the main intersection, and the first phase green time should not be less than the maximum value of the north inlet straight lane clearing time, i.e., the first phase green time should satisfy the following constraints:

3 $${g_1} \geqslant \frac{{3600 \times {Q_{ns}}}}{{{m_1} \times n \times {S_{ns}}}}$$

Where: ${g_1}$ is the green time of the first phase/s; ${Q_{ns}}$ is the north inlet straight-ahead traffic volume/pcu; ${m_1}$ is the number of north inlet straight lanes;* n* is the number of intersection signal cycles in an hour; ${S_{ns}}$ is the saturation flow rate of the north inlet straight lanes/pcu.h^− 1^.

II. In order to ensure the efficiency of the main intersection, all vehicles traveling straight ahead on the west inlet should also exit the main intersection, i.e., the green time of the second phase should satisfy the following constraints:

4 $${g_2} \geqslant \frac{{3600 \times {Q_{ws}}}}{{{m_2} \times n \times {S_{ws}}}}$$

Where: ${g_2}$ is the green time of the second phase/s; ${Q_{ws}}$ is the west inlet straight-ahead traffic volume/pcu; ${m_2}$ is the number of west inlet straight lanes;* n* is the number of intersection signal cycles in an hour; ${S_{ns}}$ is the saturation flow rate of the west inlet straight lanes/pcu.h^− 1^.

III. In order to reduce the number of stops for vehicles already exiting the main intersection, it should be ensured that the west inlet vehicles traveling straight through the intersection pass through the pre-signal intersection smoothly before the displaced left-turn vehicles are permitted to change lanes. Specifically, the last vehicle traveling straight through the intersection must move smoothly past the displaced left-turn lane stop line. The opening time of the roadway pre-signal is:

5 $${t_3} \geqslant \vartriangle {t_1}=\frac{{{l_j}+{l_1}}}{{{{\bar {v}}_0}}}$$

Where: ${t_3}$ is the roadway pre-signal green time on moment/s; $\vartriangle {t_1}$ is the interval between the roadway pre-signal green time on moment and the end of the yellow light of the second phase/s; ${l_j}$ is the length of the path for westbound straight vehicles traveling within the main intersection/m; ${l_1}$ is the length of displaced left-turn lane /m; ${\bar {v}_0}$ is the traveling speed of the last straight vehicle from the west inlet/m.s^− 1^.

IV. Furthermore, in order to avoid queuing vehicles from blocking the main intersection and affecting the intersection’s traffic efficiency and safety, the roadway pre-signal should be closed ahead of time when west inlet straight vehicles pass through the main intersection in second phase. Which ensures that the left-turn queuing vehicles of the north inlet are completely dispersed, allowing the west inlet straight vehicles to pass through the displaced left-turn lane consecutively.

6 $${t^{\prime}_3}={g_1} - \vartriangle {t_2}+A$$

Where: ${t^{\prime}_3}$ is off time of the pre-signal/s; ${g_1}$ is the green time for first phase/s;$\vartriangle {t_2}$ is the interval between the green light opening moment of the second phase and the end moment of the yellow light of the roadway pre-signal;* A* is the yellow time/s.

7 $$\vartriangle {t_2}=N \times {\bar {t}_h} - \frac{{{l_j}}}{{{{\bar {v}}_1}}}$$

Where:* N* is the number of vehicles queuing for left-turn at the north inlet; ${\bar {t}_h}$ is the average headway of left-turning vehicles driving through the displaced left-turn lane stop line at the north inlet/s; ${l_j}$ is the length of the path traveled by west inlet straight vehicles in the main intersection/m; ${\bar {v}_1}$ is the average speed of west inlet straight-ahead vehicles/m.s^− 1^.

V. In order to avoid the impact of displaced left-turn vehicles queuing at the roadway stop line to the next intersection due to the unreasonable design of the roadway pre-signal, the green time of the roadway pre-signal shall meet the following constraints:

8 $${g_3}={t^{\prime}_3} - {t_3} \geqslant \frac{{3600 \times {Q_{el}}}}{{{m_3} \times n \times {S_{el}}}}$$

Where: ${g_3}$ is the green time of the roadway pre-signal/s; ${Q_{el}}$ is the traffic flow in the displaced left-turn lane/pcu; ${m_3}$ is the number of displaced left-turn lanes;* n* is the number of signal cycles at the intersection in an hour; ${S_{el}}$ is the saturation flow rate of the displaced left-turn lane/pcu.h^− 1^.

## Signal optimization control model for displaced left-turn intersections based on stopping delay

Due to the installation of the displaced left-turn lane, north inlet left-turn vehicles can continuously pass through the intersection stop line and stop and wait at the displaced left-turn lane stop line, which can be viewed as the stop line displaced forward and the signal control displaced back. The displaced left-turn vehicles wait at the roadway stop line and can continuously pass through the intersection after being released, which can be viewed as the stop line moving back and the signal control moving forward, and the delays of the north inlet left-turn vehicles and the east inlet displaced left-turn vehicles are moved to the roadway pre-signal. Therefore, the motor vehicle delay model for displaced left-turn intersections established in this paper includes two parts: the main signal delays at the intersection and the pre-signal delays at the pre-signal intersection.

### Main signal delays

A dedicated right turn lane is provided at the west inlet of the one two-way and three one-way traffic intersection, where right-turning vehicles can leave the intersection directly with negligible delay in the improved displaced left-turn traffic organization. With a dedicated left-turn lane on the north inlet, left-turning vehicles on the north inlet can leave the intersection directly without traffic signal control, and their delay at the intersection is negligible. Therefore, the main signal delays at the intersection is mainly the delay of the north inlet straight vehicles and the west inlet straight vehicles. The calculation of the average delay for these vehicles can be performed using Webster’s delay model^18^.

I. The average vehicle delay for vehicles traveling straight ahead the north inlet is:

9 $${d_{ns}}=\frac{{c \times {{(1 - {\lambda _{ns}})}^2}}}{{2(1 - {y_{ns}})}}+\frac{{{x_{ns}}^{2}}}{{2{q_{ns}} \times (1 - {x_{ns}})}} - 0.65{(\frac{c}{{{q_{ns}}^{2}}})^{\frac{1}{3}}} \times {x_{ns}}^{{(2+5{\lambda _{ns}})}}$$

Where: ${d_{ns}}$ is the average delay of the north inlet straight vehicles/s.pcu^− 1^;* c*is the cycle time/s; ${\lambda _{ns}}$ is the green ratio of the north inlet straight phase; ${y_{ns}}$ is the flow ratio of the north inlet straight phase; ${x_{ns}}$ is the saturation of the north inlet straight phase; ${q_{ns}}$ is the arrival rate of the north inlet straight vehicles/pcu.s^− 1^.

II. The average vehicle delay for vehicles traveling straight ahead the west inlet is:

10 $${d_{ws}}=\frac{{c \times {{(1 - {\lambda _{ws}})}^2}}}{{2(1 - {y_{ws}})}}+\frac{{{x_{ws}}^{2}}}{{2{q_{ws}} \times (1 - {x_{ws}})}} - 0.65{(\frac{c}{{{q_{ws}}^{2}}})^{\frac{1}{3}}} \times {x_{ws}}^{{(2+5{\lambda _{ws}})}}$$

Where: ${d_{ws}}$ is the average delay of the west inlet straight vehicles/s.pcu^− 1^;* c* is the cycle time/s; ${\lambda _{ws}}$ is the green ratio of the west inlet straight phase; ${y_{ws}}$ is the flow ratio of the west inlet straight phase; ${x_{ws}}$ is the saturation of the west inlet straight phase; ${q_{ws}}$ is the arrival rate of the west inlet straight vehicles/pcu.s^− 1^.

### Roadway pre-signal delays

According to the coordinated control of the main and pre-signal, when the west inlet straight ahead vehicles are released, the north inlet left-turn queue vehicles have all dissipated, and the west inlet straight-ahead vehicles can pass through the displaced left-turn lane stop line continuously with negligible delay at the roadway pre-signal. Therefore, the roadway pre-signal delays are mainly displaced left-turn vehicles delay and north inlet left-turn vehicles delay. Based on their characteristics, the average vehicle delay of displaced left-turn vehicles and the average vehicle delay of north inlet left-turn vehicles can also be calculated using the Webster model^18^.

I. The average vehicle delay for pre-signal displaced left-turn vehicles is:

11 $${d_{el}}=\frac{{c \times {{(1 - {\lambda _{el}})}^2}}}{{2(1 - {y_{el}})}}+\frac{{{x_{el}}^{2}}}{{2{q_{el}} \times (1 - {x_{el}})}} - 0.65{(\frac{c}{{{q_{el}}^{2}}})^{\frac{1}{3}}} \times {x_{el}}^{{(2+5{\lambda _{el}})}}$$

Where: ${d_{el}}$ is the average delay of the roadway pre-signal displaced left-turn vehicles/s.pcu^− 1^;* c* is the cycle time/s; ${\lambda _{el}}$ is the green ratio of the roadway pre-signal displaced left-turn phases; ${y_{el}}$ is the flow ratio of the roadway pre-signal displaced left-turn phases; ${x_{el}}$ is the saturation of the roadway pre-signal displaced left-turn phases; ${q_{el}}$ is the arrival rate of the roadway pre-signal displaced left-turn vehicles/pcu.s^− 1^.

II. The average vehicle delay for left-turning vehicles on the pre-signalized north inlet is:

12 $${d_{nl}}=\frac{{c \times {{(1 - {\lambda _{nl}})}^2}}}{{2(1 - {y_{nl}})}}+\frac{{{x_{nl}}^{2}}}{{2{q_{nl}} \times (1 - {x_{nl}})}} - 0.65{(\frac{c}{{{q_{nl}}^{2}}})^{\frac{1}{3}}} \times {x_{nl}}^{{(2+5{\lambda _{nl}})}}$$

Where: ${d_{nl}}$ is the average delay of the roadway pre-signal north inlet left-turn vehicles/s.pcu^− 1^;* c* is the cycle time/s; ${\lambda _{nl}}$ is the green ratio of the left-turn north inlet and the straight ahead phase of the west inlet of the roadway signal; ${y_{nl}}$ is the flow ratio of the left-turn north inlet and the straight ahead phase of the west inlet of the roadway pre-signal; ${x_{nl}}$ is the saturation of the left-turn north inlet and the straight ahead phase of the west inlet of the roadway pre-signal; ${q_{nl}}$ is the arrival rate of the north inlet left-turn vehicles of the roadway pre-signal/pcu.s^− 1^.

### Optimal control model for displaced left-turn intersection signal

With the intersection average vehicle delay minimization as the optimization objective, the displaced left-turn signal optimal control model is:

13 $$\hbox{min} d=\frac{{\sum {{D_i}} }}{{{Q_{ns}}+{Q_{ws}}+{Q_{el}}+{Q_{nl}}}}$$

Where:* d* is the average vehicles delay of the displaced left-turn intersection/s.pcu^− 1^;* i* is the signal phase, where *i* = 1,2,3,4 representing the north inlet straight phase, west inlet straight phase, displaced left-turn phase, the north inlet left-turn and the west inlet straight phase; ${D_i}$ is the delay for the i-th phase of the vehicles/s; ${Q_{ns}}$ is the north inlet straight flow/pcu; ${Q_{ws}}$ is the west inlet straight flow/pcu; ${Q_{el}}$ is the displaced left-turn flow/pcu; ${Q_{nl}}$ is the north inlet left-turn flow/pcu.

The optimized control model for displaced left-turn signal at intersection should satisfy the following constraints:

14 $$c={g_1}+{g_2}+{l_{loss}}$$

15 $${g_{1\hbox{min} }} \leqslant {g_1} \leqslant {g_{1\hbox{max} }}$$

16 $${g_{2\hbox{min} }} \leqslant {g_2} \leqslant {g_{2\hbox{max} }}$$

Equation (14), Eq. (15), Eq. (16):* c* is the cycle time/s; ${g_1}$, ${g_2}$ are the green time for the first and second phases/s; ${l_{loss}}$ is the cycle loss time/s; ${g_{1\hbox{min} }}$, ${g_{2\hbox{min} }}$ are the shortest green time for the first and second phases/s, that is, to meet the minimum pedestrian crossing time; ${g_{1\hbox{max} }}$, ${g_{2\hbox{max} }}$ is the longest green light time for the first and second phases/s.

The variables to be optimized in this model include the signal cycle time *c*, the phase green time ${g_1}$,${g_2}$, and the signal cycle time and the phase green time are both positive integers. It is a nonlinear integer optimization model. In this paper, the Monte Carlo search method is used to obtain the approximate solution of the model.

## Case studies

### Intersection status analysis

The intersection of Haihe Road and Xuanqing Street in Harbin, China, was a cross-shaped intersection. Xuanqing Street implements one-way traffic for vehicles traveling from north to south, Pinggong Street implements one-way traffic for vehicles traveling from west to east, and Haihe Road implements two-way traffic. Specifically, the north inlet of the intersection prohibits right turns, the west inlet prohibits left turns, and the east inlet prohibits straight and right turns. The current channelization form is shown in Fig. 8a.

**Fig. 7:**
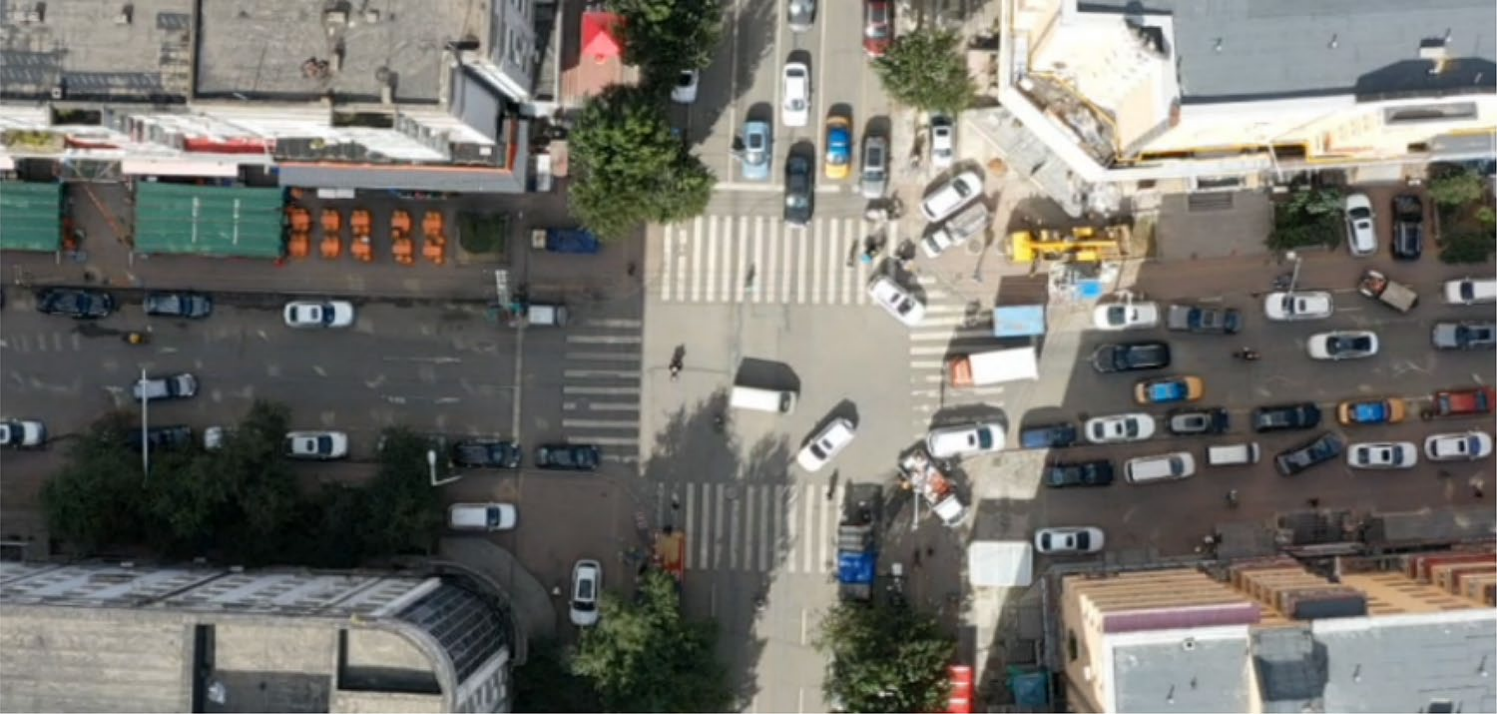
Drone aerial view of the surveyed intersection.

**Fig. 8:**
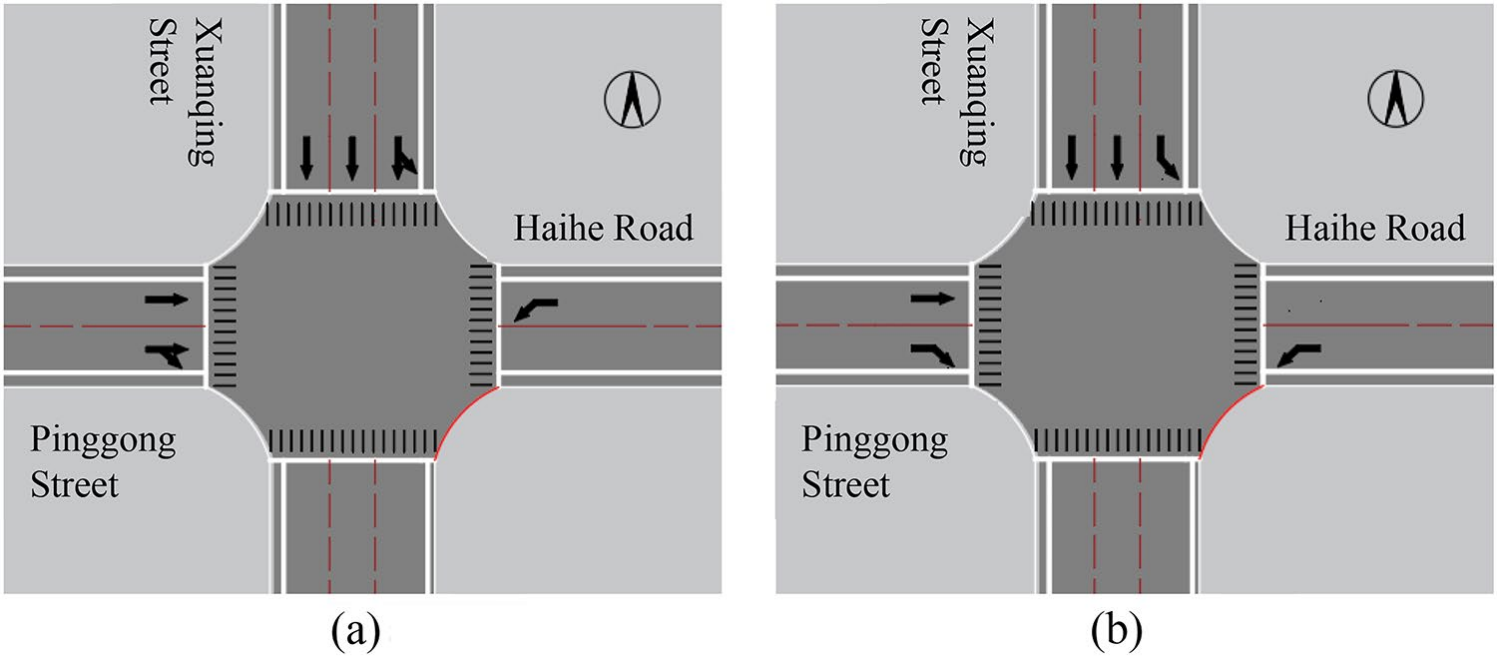
Comparison of current channelization and displaced left-turn design channelization at the intersection.(**a**) Investigating the currentchannelization of intersection, (**b**) Displaced left-turn intersectionchannelization

Traffic surveys were conducted at the intersection of Haihe Road and Xuanqing Street in Harbin, China, during the morning peak hour (7:10 − 8:10 a.m.) using drones for multiple consecutive days. The overhead image of the intersection is shown in Fig. 7. The intersection signal control mode during the left-turn permit phase allowed both left-turn and straight traffic to be released simultaneously. Left-turn vehicles stopped and yielded for the opposite direction of the straight traffic to pass before proceeding, or they looked for gaps in the opposite direction of the straight traffic to traverse the intersection. Specifically, the first phase was for the north inlet straight and left turns, and the second phase was for the west inlet straight and east inlet left turns. This study aims to determine the optimal efficiency of intersection traffic operation by selecting the most unfavorable data from multiple days, namely the morning peak hour data with the most severe conflict between straight vehicles at the west entrance and left turning vehicles at the east entrance. Among them, the hourly traffic flow on the west inlet was 541 pcu/h for straight traffic and 520 pcu/h for right-turn traffic; the hourly traffic flow on the north inlet was 1,561 pcu/h for straight traffic and 449 pcu/h for left-turn traffic; the hourly traffic flow on the east inlet was 518 pcu/h for left-turn traffic.

According to the peak hour traffic volume, the intersection had a high traffic density per unit time, with a peak hour traffic flow of 3,589 pcu/h. The west inlet right-turn flow accounted for about half of the total flow of the west inlet, and the north inlet left-turn vehicles accounted for a larger proportion. Based on the current signal time of the intersection, the cycle time was 78s, with the green time of the first phase being 38s, the green time of the second phase being 34s, and the yellow time being 3s. The collected traffic data was loaded into the VISSIM software for traffic simulation, and the simulated data was output using the VISSIM software. The output included the average vehicles delay at the intersection, the average delay of vehicles at each inlet, the average stopping time, the average queue length, and the maximum queue length, as shown in Table 1.

**Table 1:** Signal control simulation results for the status Quo intersection.

Vehicle routing	average vehicles delay(s)	average stopping time(s)	average queue length(m)	maximum queue length(m)
West inlet straight ahead	35.23	25.19	68.86	241.91
North inlet straight ahead	14.12	10.77	35.78	139.82
North inlet turn left	16.32	11.92	31.18	98
East inlet turn left	134.97	75.95	163.51	367.35
intersection	28.61	19.42		

### Displaced left-turn lane setup and regional traffic organization design

Firstly, the displaced left-turn lane was set up for the east approach of the Haihe Road-Xuanqing Street intersection. The left-turn traffic volume from the north inlet accounted for a significant proportion, approximately one-quarter of the total approach volume. The conflict between straight vehicles and left-turn vehicles in the combined straight-left lane severely affected the intersection’s efficiency. Therefore, the combined straight-left lane was converted into a dedicated left-turn lane. The right-turn traffic volume from the west inlet was relatively high, accounting for about half of the total approach volume. To reduce the interference of straight vehicles on right-turn vehicles and decrease the delay for right-turn vehicles, thereby improving the intersection’s efficiency, the combined straight-right lane on the west inlet was converted into a dedicated right-turn lane. After implementing the displaced left-turn traffic organization for this one one-way and three two-way intersection, the internal channelization of the intersection is shown in Fig. 8(b).

In addition to considering the main intersection channelization, there was a need for further design of the main signal intersection, pre-signal intersection, and regional traffic organization. The displaced left-turn signal traffic organization within the scope of influence of traffic channelization is shown in Fig. 2. Diversion lines were set up inside the intersection for left-turn vehicles at the east inlet and straight vehicles at the west inlet, which was conducive to correct driving by drivers and improved the safety of the intersection. Pre-signal, stop lines, guide lines, and a speed limit sign were also installed at the pre-signal intersection to ensure safe driving. A stop sign was set up at the left-turn lane at the north inlet of the intersection. When the straight vehicles at the west inlet of the intersection were released, the left-turning vehicles at the north inlet gave way to the straight vehicles at the west inlet to avoid blocking the intersection. Additionally, speed limit signs, pedestrian crossing lines, and other measures were implemented simultaneously.

### Calculation of intersection main and pre-signal time

**Fig. 9:**
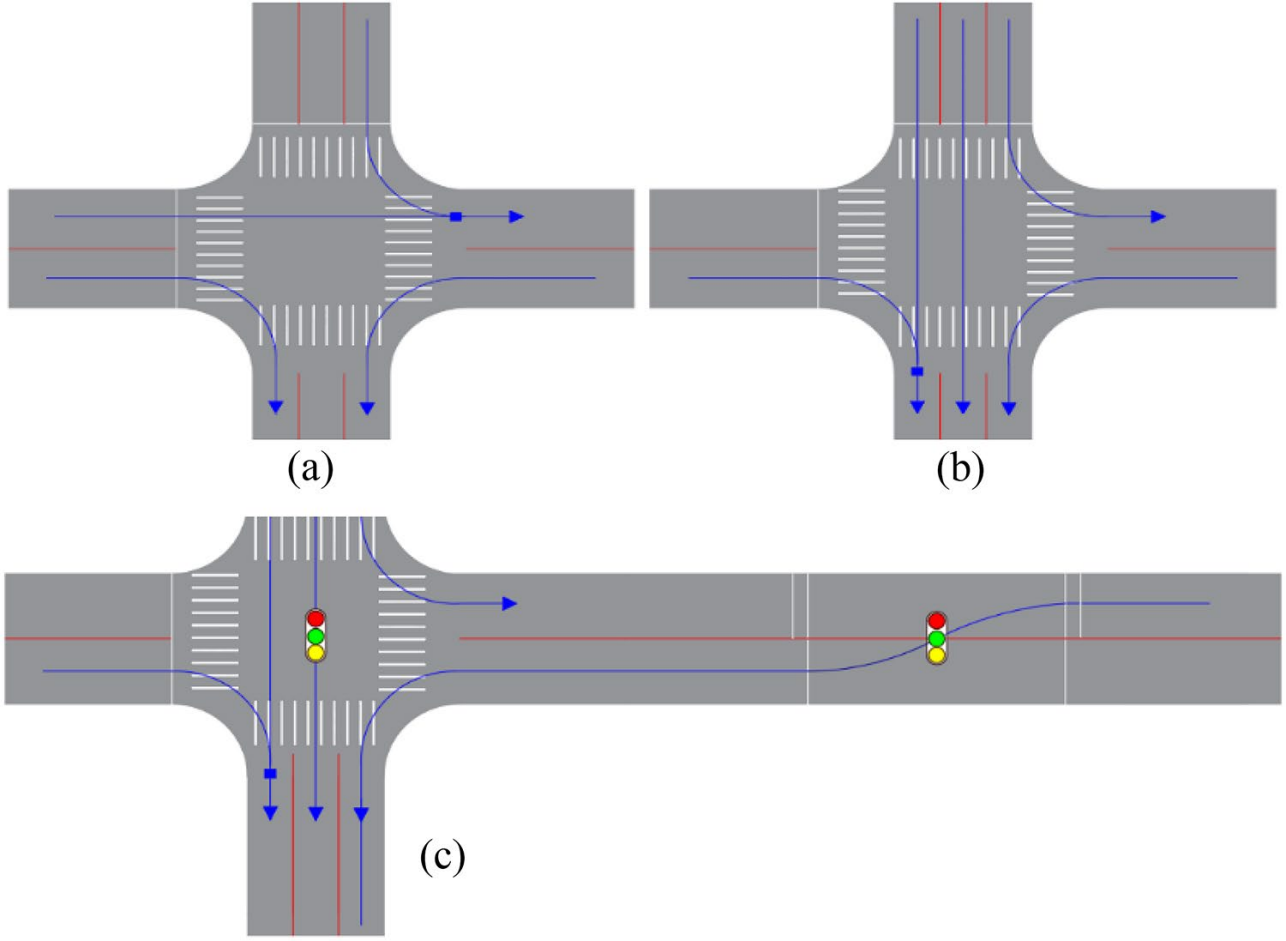
Coordinated design of main and pre-signal phase sequences for displaced left-turn intersection. (**a**) First phase of the main signal (**b**) Second phase of the main signal, (**c**) First phase of the pre-signal

Displaced left-turn vehicles utilized the rightmost lane of the south inlet, and there was no conflict with the north inlet straight-through vehicles. As a result, displaced left-turn vehicles could pass directly without signal control. Similarly, left-turning vehicles on the north inlet and right-turning vehicles on the west inlet could also pass without signal control. Therefore, to maximize the benefits of the displaced left-turn lane and the intersection, the main signal phases at the intersection were designed as two phases: the north inlet straight in the first phase and the west inlet straight in the second phase. The intersection main signal phasing design is shown in Fig. 9a, b.

In order to maximize the overall capacity of the intersection, the intersection main signal was designed in coordination with the roadway signal. When the roadway signal was on, left-turning vehicles from the east inlet drove through the roadway stop line and passed through the intersection continuously. At this time, north inlet arriving left-turn vehicles lined up in front of the displaced left-turn lane stop line. When west inlet straight-ahead vehicles were moving through, the roadway pre-signal was turned off early to allow the north inlet left-turn queuing vehicles to dissipate early, enabling the west inlet straight-ahead vehicles to pass through the displaced left-turn lane continuously. The first phase of the pre-signal was designed to coordinate with the main signal, as shown in Fig. 9c.

According to the results of the traffic survey of Haihe Road - Xuanqing Street intersection, the headway of the north inlet left-turn vehicles in the queue ${h_s}$ was 5.5 m, the speed of the last straight vehicle in the north inlet ${\bar {v}_0}$ was taken as 6.95 m/s, and the average speed of the straight vehicles in the west inlet ${\bar {v}_1}$ was 4.17 m/s, and the minimum radius of the vehicles *r* was taken as 13.3 m.

The improved motor vehicle delay model for displaced left-turn intersections was analyzed using Python with Monte Carlo search method, with the step size set to 1s and the number of iterations to 10,000, to obtain the minimum vehicles vehicles delay d of 15.25s/pcu.The signal cycle time *c* was 45s, with the display green time of the first phase being 24s, and the yellow time being 3s. the display green time of the second phase was 15s, and the yellow time was 3s. The roadway signal displayed a green time of 16s and a yellow time of 3s. Based on the signal cycle, the green time of each phases, the average vehicle, the maximum turning radius of vehicles, and the head spacing of the left-turn vehicles queuing up at the north inlet, the length of the displaced left-turn lane ${l_1}$ was determined to be 30 m, the width of the pre-signalized intersection ${l_2}$ was determined to be 18 m, and the interval between the opening of the green light of the roadway pre-signal and the end of the yellow light of the second phase $\vartriangle {t_1}$ was determined to be 3s, and the interval between the opening of the green light of the second phase and the end of the yellow light of the roadway pre-signal $\vartriangle {t_2}$ was determined to be 5s.

### Vissim simulation analysis

The optimized intersection data were loaded into Vissim software for simulation, and the node method in Vissim software was used to output the simulated data. The output included the average vehicles delay, average stopping time, average queue length, and the maximum queue length for each inlet lane of the optimized intersection, as shown Table 2.

**Table 2:** Simulation results of Optimal Control of Displaced Left-turn Organization.

Vehicle routing	Average vehicles delay(s)	Average stopping time(s)	Average queue length(m)	Maximum queue length(m)
West inlet straight ahead	14.15	6.95	11.91	53.11
North inlet straight ahead	7.60	2.87	7.76	61.12
North inlet turn left	15.11	6.51	8.46	59.42
East inlet turn left	12.40	6.78	8.96	47.06
intersection	9.93	4.37		

**Fig. 10:**
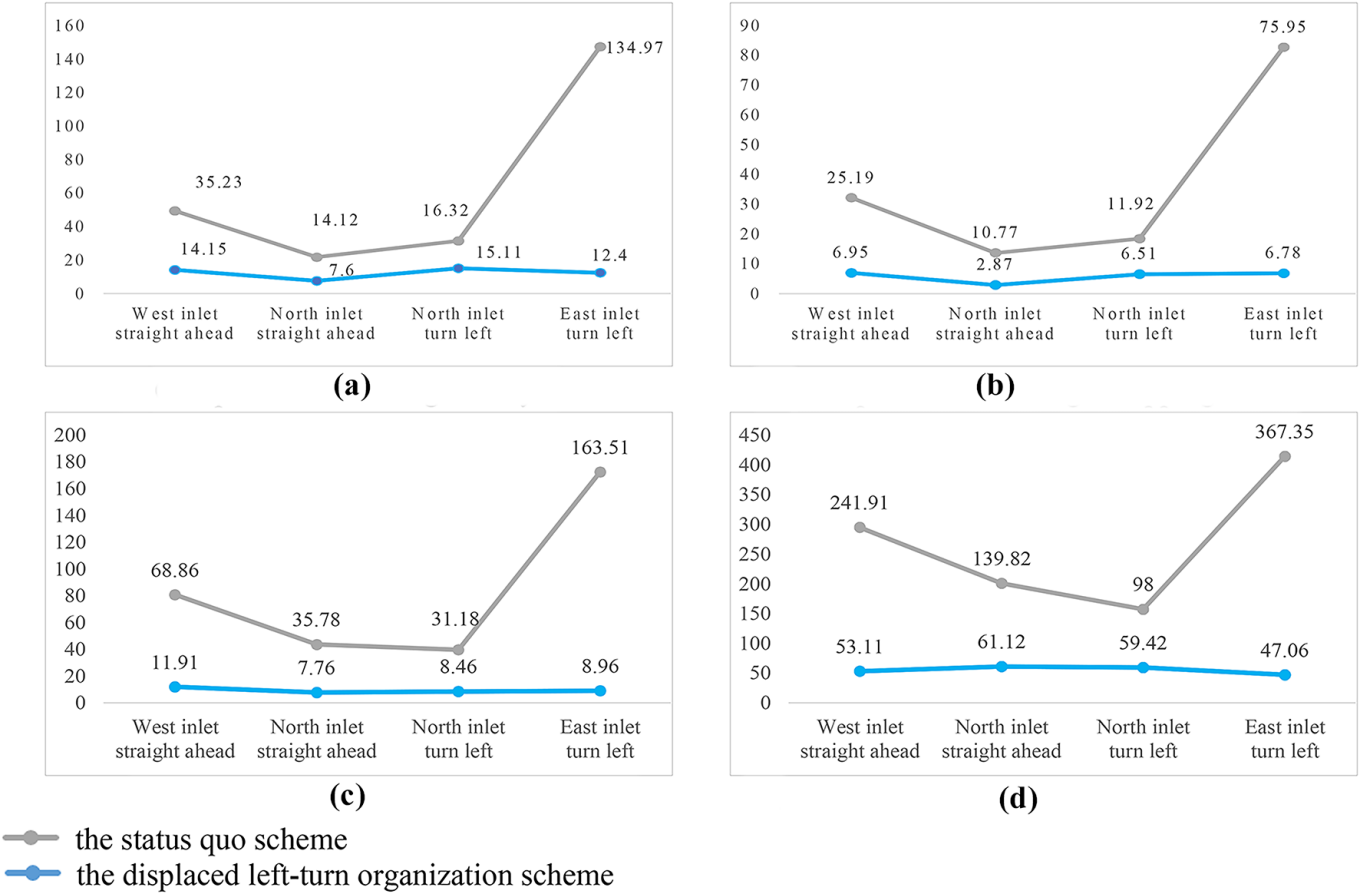
Comparative analysis of simulation results between the displaced left-turn organization scheme and the status Quo scheme.(**a**) comparison of average delays, (**b**). comparison of average stopping time, (**c**) comparison of average queue length (**d**) comparison of maximum queue length

The data from the simulation results of the current situation of the intersection and the simulation results of the optimized scheme were compared and analyzed, as shown in Fig. 10. The total average delay of the traffic flow of the intersection before optimization was 28.61s, and the average stopping time was 19.42s. After adopting the displaced left-turn design of the intersection, there was a significant reduction in the average delay and the average stopping time, which were reduced to 9.93s and 4.37s, respectively. Notably, the efficiency of the east inlet left-turn traffic was greatly improved. The average delay of the east inlet left-turn traffic before optimization was 134.97s, the average stopping time was 75.95s, the average queuing length was 163.51 m, and the maximum queuing length was 367.35 m. After optimization, the average delay was reduced to 12.4s, the average stopping time was reduced to 6.78s, the average queuing length was reduced to 8.96 m, and the maximum queuing length was reduced to 47.06 m. Traffic flow in other directions also showed varying degrees of improvement. It can be seen that the setup of the displaced left-turn lane can improve the capacity of the one two-way and three one-way traffic intersections effectively, significantly reducing traffic delay and the number of vehicle stops in single-double traffic intersections, with substantial traffic benefits.

## Conclusions

In this study, displaced left-turn lanes were applied to a single-double traffic intersections for the first time, and a signal optimization control model aimed at minimizing average vehicle delay was established through designing the length of displaced left-turn lanes, determining the length of pre-signal intersection, and coordinating between the pre-signal and the main signal. An in-depth analysis was conducted on the one two-way and three one-way traffic intersection of Haihe Road-Xuanqing Street in Harbin, China, and the effectiveness of the scheme was verified by using Vissim simulation tool. The results show that the displaced left-turn organization improves the intersection operation efficiency by further reducing the conflict points at single and double traffic intersections, and also provides an effective solution for similar complex traffic environments, which is particularly applicable to the peak hour management of high-capacity single-double traffic intersections.

Several areas require further enrichment and improvement in future research:

1. This paper only designed and verified the feasibility of an example of one two-way and three one-way traffic intersection. Future studies can extensively examine single-double traffic intersections with different flow rates and different channelization forms.
2. Although the displaced left-turn design for single-double traffic intersections reduces the conflict points, further research and discussion are needed to enhance intersection safety.
3. This paper focused on isolated a single-double traffic intersection. In the future, the scope of the study can be expanded to discuss the possibility and benefits of implementing displaced left-turn lanes in one-way traffic networks.

## Data Availability

The authors confirm that the data supporting the findings of this study are available within the article.
